# Clinicopathological features and diagnostic challenges of hepatic epithelioid angiomyolipoma: a retrospective analysis of 27 cases

**DOI:** 10.3389/fmed.2026.1729519

**Published:** 2026-02-09

**Authors:** Erming Cai, Hongzhen Wei, Yizhuo Shi, Shiran Sun, Chidan Wan

**Affiliations:** Department of Hepatobiliary Surgery, Union Hospital, Tongji Medical College, Huazhong University of Science and Technology, Wuhan, China

**Keywords:** clinicopathology, diagnosis, hepatic epithelioid angiomyolipoma, immunohistochemistry, malignant potential

## Abstract

**Background:**

Hepatic epithelioid angiomyolipoma, a rare mesenchymal tumor, poses significant diagnostic challenges due to its rarity and non-specific presentation. Existing literature is dominated by case reports, limiting a systematic understanding of its clinicopathological profile. Therefore, further in-depth research is of great significance for elucidating the nature and clinical features of this disease.

**Objective:**

To define the clinicopathological characteristics, diagnostic features, and management outcomes of Hepatic epithelioid angiomyolipoma by analyzing a single-center cohort.

**Methods:**

We retrospectively analyzed 27 patients diagnosed with hepatic epithelioid angiomyolipoma at a single institution between April 2019 and November 2024. We collected and examined data on clinical presentation, laboratory findings, imaging results from ultrasound, computed tomography, and magnetic resonance imaging, as well as histopathological and immunohistochemical features.

**Results:**

The cohort was predominantly female (88.9%; 24/27), with a mean age of 44.2 years, and most patients (81.5%; 22/27) were asymptomatic. Preoperative imaging frequently mimicked other liver tumors, resulting in a correct preoperative diagnosis in only 3.7% of cases. Pathologically, all tumors tested were positive for HMB-45 (100%) and Melan-A (100%). Surgical resection was the primary treatment (96.3%). Tissue biopsy combined with microwave ablation was performed in 3.7% (1/27) of the patients. 27 cases were diagnosed as typical hepatic epithelioid angiomyolipoma.

**Conclusion:**

Hepatic epithelioid angiomyolipoma presents with non-specific clinical and radiological features, making preoperative diagnosis challenging and often leading to misdiagnosis as other common liver tumors. Definitive diagnosis depends on histopathological and immunohistochemical analysis. Surgical resection remains the primary treatment due to the tumor’s malignant potential. A high index of suspicion and reliance on pathological confirmation are crucial for accurate management of this rare tumor.

## 1 Introduction

Angiomyolipoma (AML) is a rare mesenchymal tumor that most frequently occurs in the kidney and was first described in the literature in 1951. In 1992, Bonetti et al. introduced the concept of the perivascular epithelioid cell, providing new insights into the histogenesis of AML (1). However, its primary hepatic counterpart, specifically classified as the epithelioid variant (hepatic epithelioid angiomyolipoma, HEAML), is exceedingly uncommon. Current reports on HEAML remain limited both domestically and internationally, with the majority presented as isolated case reports. Epithelioid angiomyolipoma was first formally recognized as a distinct entity in the 2004 WHO Classification of Renal Tumours (2). According to the current WHO Classification of Digestive System Tumors (5th edition, 2019), hepatic angiomyolipoma belongs to the perivascular epithelioid cell neoplasm (PEComa) family and is categorized as Angiomyolipoma, Angiomyolipoma of uncertain malignant potential, or Malignant PEComa based on histological features (3). The term “epithelioid angiomyolipoma” (EAML) continues to be used to describe tumors with predominant epithelioid morphology. Hepatic epithelioid angiomyolipoma (HEAML) is a rare mesenchymal neoplasm with potential for malignant behavior.

Owing to the absence of characteristic clinical manifestations and non-specific features on conventional imaging, the preoperative diagnosis of HEAML is challenging and associated with a high misdiagnosis rate. It is often misidentified as other hepatic tumors, such as hepatocellular carcinoma (HCC), focal nodular hyperplasia (FNH), or hepatic adenoma. This study was designed to test the hypothesis that the clinicopathological and imaging features of HEAML are non-specific, leading to a high rate of preoperative misdiagnosis. By retrospectively analyzing the clinical data of 27 HEAML patients from our institution—including their imaging presentations, pathological characteristics, and management processes—we aim to summarize its clinicopathological features and provide insights to facilitate more accurate diagnosis and optimized treatment strategies.

## 2 Materials and methods

### 2.1 Patient selection

This single-center, retrospective clinical study was conducted in the Department of Hepatobiliary Surgery at Union Hospital, Tongji Medical College, Huazhong University of Science and Technology. A total of 33 patients with pathologically confirmed hepatic epithelioid angiomyolipoma (HEAML), who were admitted between April 2019 and November 2024, were enrolled. The inclusion criteria were as follows: (1) adult patients aged ≥ 16 years; (2) definitive histopathological diagnosis of HEAML Pathological diagnosis is established based on surgically resected or biopsy specimens. The diagnosis of hepatic epithelioid angiomyolipoma (EAML) adheres to widely accepted histological criteria: the tumor is predominantly composed of epithelioid cells (> 50%), with an adipocyte component typically constituting less than 10%. All cases were confirmed by immunohistochemistry, demonstrating co-expression of melanocytic markers (such as HMB-45 and Melan-A) and smooth muscle markers (such as SMA); and (3) availability of complete clinical follow-up data. The exclusion of metastatic tumors was performed. As a retrospective study, it was strictly conducted in accordance with medical ethical standards. Written informed consent for surgical procedures and pathological examination was obtained from all patients or their legal guardians. All clinical data were anonymized to ensure patient privacy protection, complying with medical ethics regulations.

### 2.2 Clinical data and imaging materials

Complete clinical data for all enrolled patients were systematically collected. This included demographic characteristics (age, sex), clinical symptoms, medical history, and laboratory parameters (complete blood count, liver function tests, serological markers for hepatitis viruses, and tumor markers). Detailed tumor morphological characteristics (size, anatomical location) and histopathological diagnostic results (including routine pathology and immunohistochemistry data) were also recorded. For radiological evaluation, all patients underwent at least one of the following comprehensive imaging examinations: conventional hepatobiliary ultrasonography, contrast-enhanced ultrasound (CEUS), multiphasic multidetector computed tomography (CT), magnetic resonance imaging (MRI) with Primovist (Gd-EOB-DTPA) enhancement, or 18F-fluorodeoxyglucose positron emission tomography/computed tomography (18F-FDG PET/CT) metabolic imaging.

### 2.3 Pathology and immunohistochemistry

Tumor tissue specimens were fixed in 4% paraformaldehyde, routinely embedded in paraffin, and sectioned at a thickness of 4 μm. All sections were subjected to histopathological examination with hematoxylin and eosin (H&E) staining. Immunohistochemical (IHC) analysis was performed using antibodies against the following markers for systematic differential diagnosis: melanocytic markers (HMB-45 and Melan-A), mesenchymal markers [smooth muscle actin (SMA) and Desmin], a neural marker (S-100), a vascular marker (CD34), an epithelial marker [pancytokeratin (PCK)], a proliferation marker (Ki-67), and markers related to hepatocellular differentiation [alpha-fetoprotein (AFP) and Glypican-3). The Ki-67 proliferation index was assessed by eyeballing. For each case, pathologists first identified the areas with the highest density of Ki-67-positive nuclei (“hot spots”) at low magnification (scanning power 100 × ). Subsequently, at high magnification (400×), the percentage of Ki-67-positive tumor cell nuclei was estimated relative to the total number of tumor cells within these hot spot areas. The estimation was performed by experienced pathologists and reported as an approximate percentage (e.g., <5%, 5–20%, >20%). All assessments were independently reviewed by at least two pathologists, and discrepancies were resolved through consensus discussion.

### 2.4 Clinical follow-up

Follow-up was conducted through regular outpatient visits or telephone interviews, with the final follow-up date being December 2025. According to the follow-up protocol, patients were required to undergo comprehensive assessments at least every 6 months during the first postoperative year. These assessments included hepatobiliary ultrasound and liver function tests. Enhanced CT or MRI was further performed if clinically indicated. Subsequently, patients entered a long-term follow-up phase, during which they were required to complete at least one standardized annual follow-up (either an outpatient review or a structured telephone interview) to evaluate and monitor tumor recurrence and distant metastasis.

### 2.5 Statistical analysis

All statistical analyses were performed using SPSS software (version 20.0; IBM Corp, Armonk, NY, United States). Continuous variables with a normal distribution are presented as the mean ± standard deviation ($\bar{\text{x}}$ ± s), while non-normally distributed data are expressed as the median (range). Categorical variables are described as numbers (proportions, %). All statistical tests were two-sided, and a *P* < 0.05 was considered statistically significant.

## 3 Results

### 3.1 Clinical characteristics and preoperative laboratory findings

A total of 32 patients were initially identified, of whom 27 met the inclusion criteria and completed follow-up (3 males and 24 females); 4 patients were excluded due to loss to follow-up or incomplete data, Exclusion of a case initially suspected to be a metastatic malignant hepatic epithelioid angiomyolipoma of renal origin. The mean age of the patients was 44.18 ± 13.15 years (range: 17–75 years). Regarding clinical presentation, only 4 of the 27 patients (14.8%) reported intermittent upper abdominal pain or discomfort. The majority (22/27, 81.5%) were asymptomatic, with tumors discovered incidentally during routine physical examinations or investigations for other conditions. The remaining one case (3.7%) initially presented with non-specific symptoms, and a liver mass was subsequently identified following medical evaluation prompted by proteinuria accompanied by bilateral lower limb edema. No significant positive signs were found on specialized physical examination in any patient. Concerning tumor characteristics, 27 cases (100.0%) presented as a solitary lesion. The tumor diameter ranged from 1.52 to 9.8 cm (mean: 4.22 cm). The lesions were located in the right lobe in 16 cases (59.3%), the left lobe in 7 cases (25.9%), and the caudate lobe in 4 cases (14.8%). Regarding medical history, 3 patients had a history of hepatobiliary surgery, 1 had a history of teratoma resection All patients underwent routine preoperative hematological testing. Complete blood count results indicated varying degrees of anemia in 8 patients (29.6%), while the remaining 19 patients (70.2%) had parameters within the normal range. Liver function tests revealed elevated bilirubin levels in 4 patients (14.8%) and abnormal liver enzymes in 2 patients (7.4%). Serological testing for hepatitis viruses showed that 5 patients (18.5%) were positive for hepatitis B surface antigen (HBsAg); no cases were positive for hepatitis C virus antibody. Tumor marker assays demonstrated elevated alpha-fetoprotein (AFP) levels in 1 patient (3.7%), elevated carbohydrate antigen 125 (CA125) in 1 patient (3.7%), elevated carbohydrate antigen 199 (CA199) in 1 patient (3.7%), and elevated prothrombin induced by vitamin K absence or antagonist-II (PIVKA-II) in 1 patient (3.7%).

### 3.2 Preoperative imaging findings

**FIGURE 1:**
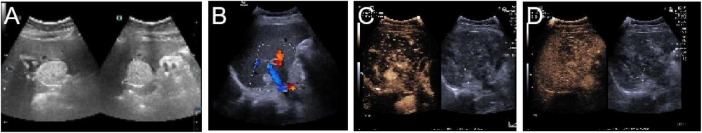
Ultrasonography and contrast-enhanced ultrasound (CEUS) findings in selected patients. **(A)** A mildly hyperechoic mass, measuring approximately 51 × 45 × 42 mm, is observed in the left lobe extending to the caudate lobe. The lesion demonstrates well-defined borders and heterogeneous internal echogenicity. **(B)** A well-defined, mildly hyperechoic and vascularized solid mass, measuring approximately 17 × 15 mm, is identified in segment VIII of the right hepatic lobe. **(C,D)** CEUS imaging reveals rapid hyperenhancement of the lesion during the arterial phase, followed by washout to isoenhancement in the portal venous phase, and mild hypoenhancement in the delayed phase.

Four patients underwent conventional ultrasonography (US), all of which revealed solid lesions. Among these, two lesions presented as hypoechoic masses and one as a mildly hyperechoic mass. Three patients subsequently underwent contrast-enhanced ultrasound (CEUS). The CEUS findings were characterized by rapid hyperenhancement of the lesions during the arterial phase, followed by washout to isoenhancement in the portal venous phase. In the delayed phase, lesion enhancement further decreased, appearing significantly lower than that of the surrounding normal parenchyma. None of the ultrasonographic examinations in this cohort yielded an accurate preoperative diagnosis of HEAML. Figure 1 shows the ultrasound and contrast-enhanced ultrasound manifestations of a patient.

Sixteen patients underwent CT examination. On non-contrast scans, 16 lesions (100.0%) appeared as hypodense or mildly hypodense. Enhancement characteristics on contrast-enhanced scans revealed marked enhancement in all cases (16/16, 100%) during the arterial phase. This enhancement was heterogeneous in 7 cases (43.8%) and homogeneous in 1 case (6.3%). In the portal venous phase, the degree of enhancement decreased in 15 cases (93.8%) and increased in one case (6.3%). Regarding the CT-based radiological diagnosis, hepatocellular carcinoma (HCC) or small HCC was suspected in 6 cases (37.5%). A neoplastic lesion (unspecified nature) was reported in 9 cases (56.3%). Only one case (6.3%) was considered highly suggestive of a fat-poor angiomyolipoma, although a malignant tumor could not be entirely excluded.

**FIGURE 2:**
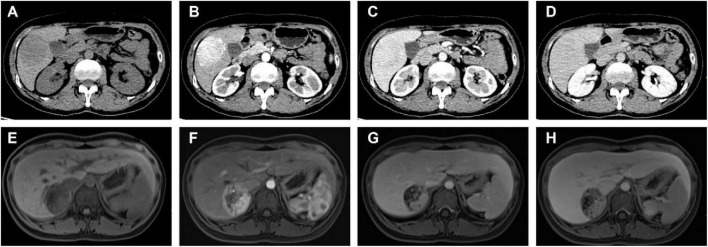
Representative CT and MRI findings from some patients. **(A–D)** CT images of a 51-year-old female patient: **(A)** Non-contrast scan shows a roundish, slightly hypodense mass in the inferior segment of the right hepatic lobe, slightly protruding beyond the liver contour, measuring approximately 6.5 × 4.5 cm. **(B)** Arterial phase contrast-enhanced scan reveals heterogeneous marked enhancement, with a CT value of approximately 114 HU. **(C)** Portal venous phase and **(D)** delayed phase images demonstrate washout of contrast, with enhancement slightly lower than that of the normal liver parenchyma. **(E–H)** MRI images in a 17-year-old female patient. **(E)** A roundish lesion exhibiting long T1 and long T2 signals is observed in the right posterior liver lobe, measuring 48 × 42 × 46 mm (transverse × craniocaudal dimensions). **(F)** Arterial phase post-contrast imaging shows obvious heterogeneous enhancement. **(G)** Portal venous phase and **(H)** delayed phase images reveal enhancement of the solid components similar to that of the adjacent liver parenchyma.

Fifteen patients underwent MRI. On diffusion-weighted imaging (DWI), 13 lesions showed high signal intensity. On T1-weighted imaging (T1WI), these 13 lesions demonstrated low signal intensity. Among them, 10 lesions exhibited mildly high signal intensity on T2-weighted imaging (T2WI), while 3 showed high signal intensity. During the arterial phase of contrast-enhanced MRI, 9 lesions demonstrated marked enhancement, 2 showed heterogeneous mild enhancement, and 2 exhibited a “wash-in and wash-out” pattern. In the portal venous phase, 11 lesions showed washout, with the enhancement degree being lower than the surrounding liver parenchyma in 2 cases and similar in 1 case. In the delayed phase, 11 lesions exhibited decreased enhancement, with one case showing capsular enhancement. In the hepatobiliary phase, 12 lesions appeared as hypointense. Regarding the MRI-based radiological diagnosis, 10 cases were reported as neoplastic lesions (unspecified); among these, one report specifically mentioned the need for differential diagnosis between HCC and a hypervascular benign lesion such as epithelioid AML. The remaining 5 cases were diagnosed as HCC or small HCC. Table 1 summarizes and compares the detailed CT/MRI imaging features of 27 patients in this study. In this study, as shown in Table 2, only 1 cases (3.6%) were accurately diagnosed with HEAML based on imaging examinations. Figure 2 shows the representative CT and MRI manifestations of some patients.

**TABLE 1:** Summary of CT/MRI imaging characteristics in 27 patients.

Imaging items and results		Manifestation types	Variable	Percentage (%)
Computed tomography (CT) examination (*n* = 16)	Non-enhanced density	Hypodense/Mildly Hypodense	16	100.0
	Arterial phase enhancement	Heterogeneous enhancement	7	43.8
		Homogeneous enhancement	1	6.3
		Other enhancement patterns	8	50.0
	Portal Venous Phase enhancement	Washout (decreased enhancement)	15	93.8
		Increased enhancement	1	6.3
	Preliminary CT Diagnosis	Suspected HCC/Small HCC	6	37.5
		Neoplastic lesion (unspecified)	9	56.3
		Angiomyolipoma	1	6.3
Magnetic Resonance Imaging (MRI) Examination (*n* = 15)	DWI signal	High signal intensity	13	86.7
	T1WI signal	Low signal intensity	13	86.7
	T2WI signal	Mildly high signal intensity	10	66.7
		High signal intensity	3	20.0
	Arterial phase enhancement	Marked enhancement	9	60.0
		Heterogeneous mild enhancement	2	13.3
		Wash-in and wash-out	2	13.3
	Portal phase enhancement	Decreased enhancement	11	73.3
		Lower than liver parenchyma	2	13.3
		Similar to liver parenchyma	1	6.7
	Delayed Phase	Signal reduction	11	73.3
		Capsular enhancement	1	6.7
	Hepatobiliary phase signal	Low signal intensity	12	80.1
	MRI diagnosis	HCC/Small HCC	5	33.3
		Neoplastic lesion (unspecified)	10	66.7

**TABLE 2:** Comparison of preoperative imaging diagnoses.

Examination method	No. of cases examined	Diagnostic category	Variable	Percentage (%)
US	4	Neoplastic lesion	4	100.0
		HCC/small HCC	0	0.0
		HELAM	0	0.0
CEUS	3	Neoplastic lesion	2	66.7
		HCC/small HCC	1	33.3
		HELAM	0	0.0
CT	16	Neoplastic lesion	9	56.3
		HCC/small HCC	6	37.5
		HELAM	1	6.3
MRI	15	Neoplastic lesion	10	66.7
		HCC/small HCC	5	33.3
		HELAM	0	0.0

### 3.3 Pathological features and immunohistochemical staining

To establish a definitive diagnosis and guide subsequent treatment strategies, all 27 enrolled patients underwent pathological examination. Among them, 26 patients provided specimens obtained via surgical resection of the lesion, while the remaining 1 patient provided specimens acquired through intraoperative liver needle biopsy. A pathological diagnosis was confirmed in all cases. Gross specimen analysis revealed that the tumor diameter was ≤ 3 cm in 8 cases (30.8%), 3–5 cm in 12 cases (46.2%), 5–10 cm in 6 cases (23.1%). The gross characteristics of the tumors primarily presented as solid nodules with a generally moderate or slightly soft consistency. The cut surface exhibited diverse coloration: grayish-white in 6 cases, grayish-yellow in 5 cases, mixed colors (e.g., grayish-brown with red areas, grayish-white with yellow areas, grayish-yellow with brown areas) in 12 cases, and grayish-red in 2 cases; the color features were not recorded in the remaining 2 cases. Special morphological alterations identified on histological examination included cystic changes and cavity formation in 2 cases, and the presence of necrotic foci in 2 cases. Regarding tumor margin characteristics, 2 cases were well-demarcated from the surrounding liver tissue (1 case with clear margins and 1 cases with relatively clear margins), 6 cases showed ill-defined margins, and the margin status was not described in the remaining 19 cases. Table 3 summarizes the histopathological features of 27 patients.

**TABLE 3:** Analysis of histopathological characteristics.

Item	Classification/description	Variable	Percentage (%)
Specimen acquisition	Surgical resection	26	96.3
	Tissue biopsy	1	3.7
Tumor size	0–3 cm	8	30.8
	3–5 cm	12	46.2
	5–10 cm	6	23.1
Section color	Grayish-white	6	22.2
	Grayish-yellow	5	18.5
	Grayish-brown/Grayish-red/Grayish-yellow admixture	12	44.4
	Grayish-red	2	7.4
	Not described	2	7.4
Special histological changes	Cystic change	2	7.4
	Necrosis	2	7.4
Tumor border	Clear/relatively clear	2	7.7
	Ill-defined	6	22.2
	Not described	18	70.4

All 27 cases underwent immunohistochemical (IHC) staining analysis. The positive expression rates for tumor markers were as follows: HMB-45 (27/27, 100%), Melan-A (27/27, 100%), SMA (24/26, 92.3%), Ki-67 (< 5%: 12/21, 57.1%; ≥ 5%: 9/21, 42.9%), S100 (2/20, 10%), and CD34 (8/21, 38%). All examined tumor cells were negative for AFP (0/8), PCK (0/20), and Glypican-3 (0/17). Regarding the Ki-67 proliferation index, except for one case which showed a positivity rate exceeding 15%, the remaining cases had a positivity rate below 10%. Table 4 presents a detailed comparative analysis of the histopathological features of 27 patients.

**TABLE 4:** Immunohistochemical staining results (*n* = 27).

Marker	No. of cases tested	No. of positive cases	Positive rate (%)	Clinical significance
HMB-45	27	27	100.0	Markers of melanocytic differentiation
Melan-A	27	27	100.0	Markers of melanocytic differentiation
SMA	26	24	92.3	Myogenic/perivascular cell differentiation
Ki-67	21	–	–	Proliferative activity
≥5%		9	42.9	Stratification:
< 5%		12	57.1	One case > 15%, all others < 10%
S100	20	2	10.0	Neural differentiation markers (low expression)
CD34	21	8	38.0	Vascular endothelial differentiation
AFP	8	0	0.0	Exclude hepatocellular carcinoma
PCK	20	0	0.0	Exclude epithelial tumors
Glypican	17	0	0.0	Exclude HCC

**FIGURE 3:**
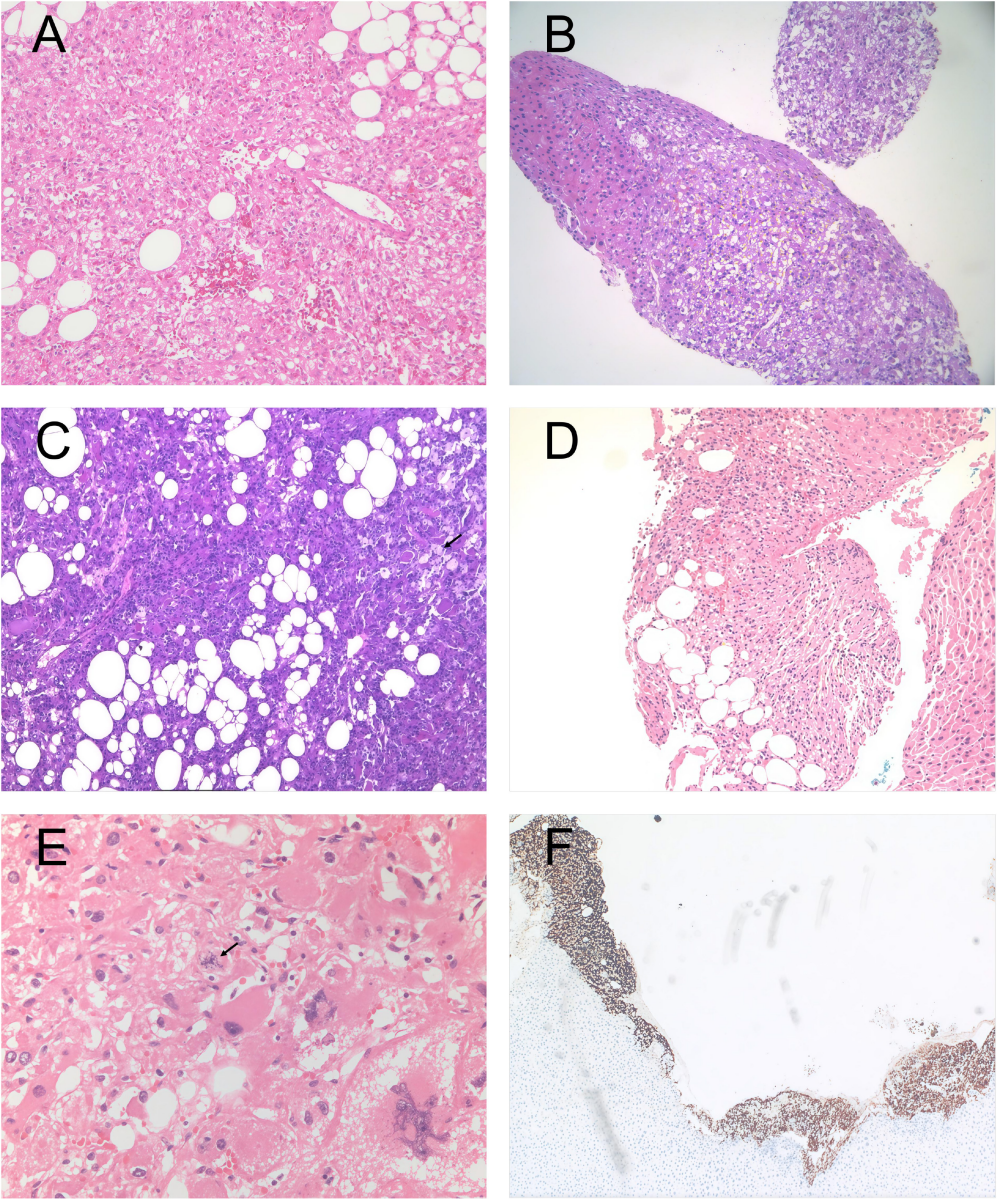
Representative histopathological and immunohistochemical findings in selected patients. **(A)** The tumor is predominantly composed of epithelioid cells with eosinophilic cytoplasm, accompanied by a small amount of adipose tissue and thick-walled blood vessels (HE staining × 100). **(B)** The tumor grows in a sheet-like pattern with relatively clear demarcation from the surrounding liver parenchyma (HE staining ×100). **(C)** Epithelioid tumor cells are arranged in nests and sheets, rich in adipose tissue, with occasional foamy cells (arrows indicate foamy cells) (HE staining × 100). **(D)** Tumor cells have abundant eosinophilic or clear cytoplasm, round or oval nuclei, and scattered adipocytes are present (HE staining × 100). **(E)** The tumor cells exhibited an epithelioid morphology with mild to moderate atypia. Mitotic figures were present, and no extensive tumor necrosis was observed (HE staining × 200). **(F)** Immunohistochemistry demonstrates diffuse positive expression of MelanA in tumor cells, with heterogeneous staining intensity.

Comprehensive histopathological and immunohistochemical analyses were performed for detailed pathological diagnosis in all 27 patients. Twenty-seven cases were diagnosed as conventional hepatic epithelioid angiomyolipoma (HEAML). As shown in the part of Figure 3, the representative histopathological and immunohistochemical results of some patients are presented. Their pathological characteristics primarily included: focal areas exhibiting typical epithelioid cell morphological features, with histopathological examination revealing that the majority of cases were predominantly composed of perivascular epithelioid cells with scarce or complete absence of adipocyte components. Immunohistochemical analysis demonstrated strong positive expression of both HMB-45 and Melan-A in all cases.

### 3.4 Treatment modalities and follow-up

**FIGURE 4:**
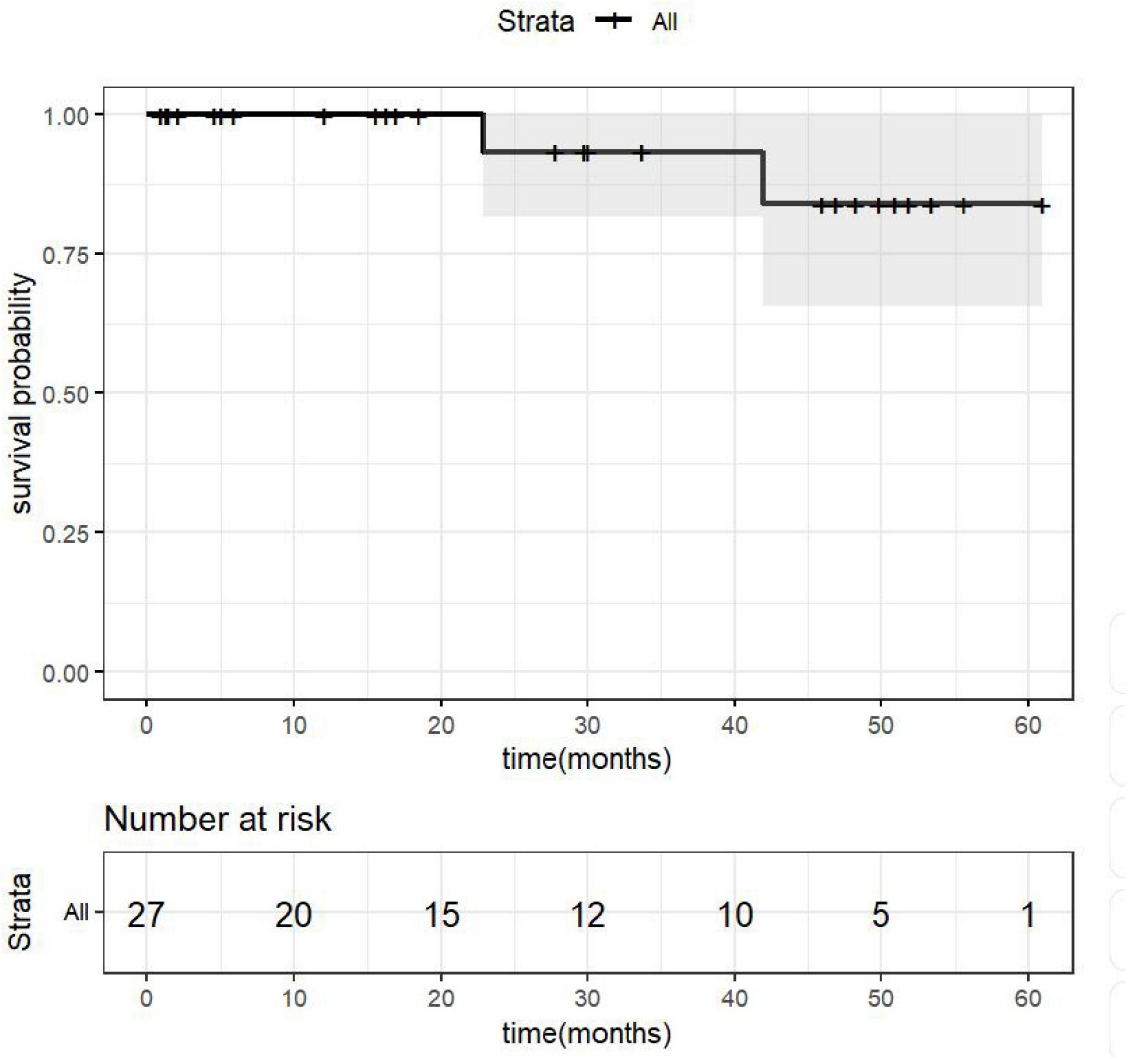
Patients’ overall survival curve.

A total of 27 patients were included in this study. Among them, 26 underwent complete laparoscopic resection of the lesions, while one patient, due to specific clinical characteristics, received laparoscopic liver tumor biopsy combined with microwave ablation. All patients recovered well after surgery and were discharged without perioperative mortality. Systematic follow-up was conducted for all patients through regular outpatient visits or telephone interviews, with the follow-up period ending in December 2025. The median follow-up time was 27.9 months (mean 25.7 months). At the time of this analysis, two patients experienced postoperative recurrence; both remained in good general condition and did not receive further treatment. No metastasis or recurrence was observed in the remaining patients. The overall survival of the patients is illustrated in the Kaplan–Meier curve shown in Figure 4. Continued follow-up will be maintained for all enrolled patients.

## 4 Discussion

Primary hepatic epithelioid angiomyolipoma (HEAML) is a rare mesenchymal tumor belonging to the perivascular epithelioid cell tumor (PEComa) family, a group of neoplasms that also includes angiomyolipoma (AML), lymphangioleiomyomatosis (LAM), and clear cell sugar tumor (CCST) of the lung (4). The nomenclature for hepatic PEComa family tumors has evolved since epithelioid angiomyolipoma was first recognized in renal tumors. The current WHO classification employs a risk-stratified approach, categorizing these tumors based on histological features rather than solely on morphological subtyping. While the term “epithelioid angiomyolipoma” remains widely used in clinical practice, current evidence emphasizes comprehensive histopathological assessment for accurate risk prediction. Although initially considered benign, a growing body of evidence now recognizes that HEAML, particularly the epithelioid-predominant variant, possesses malignant potential, creating significant diagnostic and therapeutic challenges (5, 6). Our study, which presents a series of pathologically confirmed HEAML cases, corroborates these findings and highlights the non-specific clinicopathological features that often lead to preoperative misdiagnosis, most commonly as hepatocellular carcinoma (HCC) (7, 8).

Epidemiological studies on hepatic epithelioid angiomyolipoma (HEMAL) indicate that this tumor is rare among all hepatic neoplasms, with a low annual incidence. It exhibits a significant gender predilection, with females accounting for 75–85% of cases, resulting in a male-to-female incidence ratio of approximately 1:4 (9). In the present study, the proportion of female patients reached 88.9% (24/27), further corroborating this characteristic. This disparity is hypothesized to be associated with fluctuations in estrogen levels, which may also explain the predilection of this disease for women of childbearing age (10). Existing literature reports that the age of onset for patients with hepatic angiomyolipoma primarily clusters between 30 and 50 years, with a mean age of 42 years (9, 11). Although the age distribution in our cohort was broad (range: 17–75 years), it was predominantly concentrated within the 34–52-year age range, with a mean age of 44.18 ± 13.15 years. This age distribution aligns with previous reports. HEMAL typically lacks specific clinical manifestations. A substantial majority of patients are asymptomatic, with the tumor often being discovered incidentally during imaging examinations performed for routine health check-ups or other reasons. Among symptomatic patients, a smaller subset may present with non-specific symptoms such as right upper quadrant discomfort, abdominal distension, and anorexia, which are generally attributable to the mass effect of the tumor (12). Additionally, approximately 10% of patients may have concurrent systemic diseases such as tuberous sclerosis complex, suggesting a potential link to genetic background and immune dysregulation. This association underscores the importance of vigilance for potential comorbidities during clinical evaluation (13). In our cohort of 27 HEMAL patients, 81.4% (22/27) were asymptomatic, while only 18.5% (5/27) exhibited various degrees of non-specific symptoms like abdominal pain and distension, consistent with prior reports. Overall, the non-specific clinical presentation and absence of characteristic abdominal signs contribute to the diagnostic challenge of HEMAL, resulting in high rates of misdiagnosis and missed diagnosis (14). No specific tumor markers have been definitively linked to HEMAL. In this study, only a minority of patients exhibited mild abnormalities in tumor markers: elevated alpha-fetoprotein (AFP) was observed in 1 case, elevated carbohydrate antigen 125 (CA125) in 1 case, elevated carbohydrate antigen 19–9 (CA19-9) in 1 case, and elevated protein induced by vitamin K absence or antagonist-II (PIVKA-II) in 1 case. Other common hepatic tumor markers, such as carcinoembryonic antigen (CEA), showed no significant abnormalities. With the exception of one patient who exhibited a doubling of PIVKA-II levels, all other observed marker elevations were mild to moderate and did not reach the typically extreme levels seen in hepatocellular carcinoma or cholangiocarcinoma. No concurrent diseases that could explain these marker elevations were present in these patients. Although these elevations somewhat complicated preoperative assessment, they were not considered diagnostic due to their non-diagnostic levels and atypical imaging findings, and thus were not used as definitive diagnostic criteria.

Preoperative diagnosis remains a significant hurdle due to non-specific imaging features. HEAML often mimics other hypervascular liver tumors, especially HCC. On contrast-enhanced imaging, HEAML typically shows marked arterial enhancement with subsequent washout, a pattern highly characteristic of HCC (15, 16). While the presence of intratumoral fat is a strong indicator of angiomyolipoma, it is frequently absent in epithelioid-predominant variants (lipid-poor AML), further complicating the diagnosis (17). Advanced imaging techniques, such as gadoxetic acid-enhanced MRI and contrast-enhanced ultrasound (CEUS), may offer additional clues but often fail to provide a definitive distinction from HCC or other entities like focal nodular hyperplasia (FNH) and hepatic adenoma (16, 18, 19). A substantially correct preoperative diagnosis was established by imaging in merely one case (3.6%) in this study, with the remaining cases being misdiagnosed or indeterminate. Consequently, a definitive diagnosis frequently relies on postoperative histopathology.

The histological features of hepatic epithelioid angiomyolipoma (HEAML) constitute the cornerstone of its pathological diagnosis. The typical presentation is characterized by triphasic differentiation, with the tumor tissue composed of three components: abnormal blood vessels, epithelioid smooth muscle cells, and adipocytes (20). On histological sections, HEAML typically exhibits polygonal epithelioid cells with abundant eosinophilic cytoplasm and prominent nucleoli, reflecting their metabolically active biological nature (21). In the present study, pathological sections from all 27 patients demonstrated this classical epithelioid cell morphology, consistent with previous literature. Notably, malignant HEAML may also present with specific histological variations. Luo Rongkui et al., in their clinicopathological analysis of 182 HEAML patients, summarized that features such as tumor size ≥ 10 cm, multifocal lesions, tumor invasion of the portal area, tumor necrosis, intravascular tumor thrombi, and the presence of tumor giant cells within the lesion may be closely associated with tumor recurrence or metastasis. Immunohistochemistry is the cornerstone of a definitive HEAML diagnosis. The diagnosis is confirmed by the characteristic co-expression of myoid markers (e.g., Smooth Muscle Actin, SMA) and melanocytic markers (HMB-45 and Melan-A) (22, 23). Our study confirmed consistent positivity for HMB-45 and/or Melan-A in all cases, aligning with reports that these are the most sensitive and specific markers for the PEComa family. This immunoprofile is critical for the differential diagnosis, particularly in distinguishing HEAML from its primary mimic, HCC, which is consistently negative for these melanocytic markers (24). The combined use of HMB-45 and Melan-A approaches 100% sensitivity for renal AMLs and is recommended in the workup of any suspected PEComa (23).

The core challenge of HEAML lies in its potential for aggressive behavior (25). This malignant potential is not merely a theoretical concern; a large literature review by Liu et al. (5) documented a recurrence or metastasis rate of approximately 10%, a notable figure for a tumor once considered benign. This malignant potential is underscored by specific histopathological features. Recent studies have proposed quantitative risk stratification systems incorporating multiple histopathological parameters to better predict clinical outcomes. The Folpe criteria, originally developed for extra-renal PEComas, provide a framework for risk stratification (26). Malignant PEComas are defined by the presence of two or more worrisome features, including large tumor size (> 5 cm), high mitotic rate, necrosis, and vascular invasion. In our series, several cases exhibited features such as necrosis and high nuclear grade, reinforcing the notion that HEAML is not uniformly benign and requires careful pathological assessment and long-term surveillance (27). The epithelioid component itself is a key factor; tumors composed predominantly of epithelioid cells are more likely to exhibit aggressive behavior compared to classic triphasic angiomyolipomas (28). The diagnostic criteria for HEAML vary in the literature, with epithelioid cell cutoffs ranging from 10 to ≥ 80% (29). Recent studies suggest that higher thresholds (≥ 80% epithelioid cells with minimal adipose tissue) may better correlate with malignant potential (30).

The understanding of the malignant potential of hepatic epithelioid angiomyolipoma (HEAML) has evolved beyond clinicopathological observations, progressively delving into the interplay among its cellular origins, molecular regulation, and tumor microenvironment. A hallmark feature of HEAML is its strong expression of melanocytic markers, such as HMB-45 and Melan-A, suggesting a probable origin from perivascular epithelioid cells (PECs). PECs are characterized by their bidirectional differentiation potential, giving rise to both smooth muscle and melanocytic lineages, which provides a theoretical basis for the potential malignant transformation observed in HEAML (5). At the molecular level, Studies have shown that a significant subset of PEComas harbor inactivating mutations in the TSC1 or TSC2 tumor suppressor genes (31). These mutations lead to the constitutive activation of the mammalian target of rapamycin (mTOR) signaling pathway, a key regulator of cell growth and proliferation (32). This understanding provides a strong rationale for the use of mTOR inhibitors, such as sirolimus and everolimus, in managing advanced, unresectable, or metastatic PEComas. Several studies have demonstrated the clinical activity and efficacy of these agents, establishing them as a standard of care in the systemic treatment of malignant PEComas (33). Furthermore, the malignant progression of HEAML is often accompanied by increased genomic instability. In addition to the aforementioned TSC gene mutations, multiple studies have demonstrated that abnormal expression of the P53 protein may be associated with the potential malignant behavior of HEAML (34), suggesting that its malignant transformation may involve the synergistic dysregulation of multiple pathways. Although accumulating evidence indicates a propensity for malignant transformation, well-defined diagnostic criteria for malignancy have yet to be established, posing challenges for clinical risk assessment. To address this diagnostic ambiguity, Folpe et al. (26) introduced in 2005 a three-tiered classification system (“benign,” “uncertain malignant potential,” and “malignant”) for perivascular epithelioid cell tumors (PEComas), based on a constellation of histopathological features. Key prognostic parameters in this system include tumor size exceeding 5 cm, infiltrative growth pattern, significant nuclear atypia, the presence of tumor necrosis, vascular invasion, and a mitotic index of ≥ 1 per 50 high-power fields (HPF). This provides an important framework for morphological evaluation. The malignant potential of HEAML is a biological process driven by cellular origin characteristics and specific molecular genetic events, ultimately manifesting as a series of identifiable histopathological features. A systematic assessment must go beyond mere morphological description and progress toward an integrated pathological diagnostic model that incorporates immunohistochemistry, proliferative activity, and key molecular markers. This approach enables more precise risk prediction and individualized management. In the future, multi-omics research holds promise for further elucidating the key driving mechanisms of malignant transformation and discovering novel therapeutic targets.

Given the malignant potential, surgical resection is the mainstay of treatment for localized HEAML (35, 36). Complete surgical excision offers the only potential for cure and is recommended for resectable tumors, especially if they are large, symptomatic, or if malignancy cannot be excluded (35). For patients with unresectable or metastatic disease, mTOR inhibitors are the primary systemic therapy option (32, 33). The prognosis after treatment appears to be heterogeneous. Despite successful resection, the risk of recurrence and metastasis necessitates long-term postoperative follow-up. Studies have reported metastases occurring months to years after initial surgery, emphasizing the need for vigilant surveillance (27, 35). The findings from a large 26-case series by Zhang et al. showed an excellent prognosis with no recurrence or metastasis after a median follow-up of 62.5 months, suggesting that complete resection can be highly effective (37). However, this contrasts with other reports documenting a clear risk of disease progression, highlighting the variable natural history of HEAML and reinforcing the importance of vigilant follow-up.

Despite the contributions of this study, several limitations should be noted. First, as a single-center retrospective analysis, the findings are inevitably susceptible to selection bias and information bias. All included cases were derived from a tertiary medical center, which may have resulted in the overrepresentation of patients with more complex conditions or those requiring surgical intervention. Consequently, the sample may not fully reflect the true disease spectrum of all HEAML patients in the broader community, particularly those who are asymptomatic and do not seek medical care. Second, given the rarity of hepatic HEAML, the present study involved a small sample size from a single institution, which may limit the generalizability of the results. Further investigations with larger, multicenter cohorts are necessary to obtain more robust evidence. Finally, due to the retrospective nature of this study, follow-up duration and protocols were not standardized. Although surgical resection demonstrated favorable short-term outcomes, the lack of long-term, uniformly collected follow-up data constrains our ability to accurately evaluate the risks of late recurrence and metastasis, as well as the true prognostic value of markers such as Ki-67. Future research should adopt prospective, multicenter designs and establish long-term follow-up registries to address these limitations.

## 5 Conclusion

Hepatic epithelioid angiomyolipoma (HEAML) is a rare entity whose non-specific clinical and radiological features present a significant diagnostic challenge. This study of 27 pathologically confirmed cases quantitatively underscores this challenge, revealing a preoperative diagnostic accuracy of only 3.7%, with frequent misdiagnosis as hepatocellular carcinoma. Our findings confirm that the definitive diagnosis relies on histopathology, with the consistent co-expression of HMB-45 and Melan-A being the key diagnostic feature.

Based on these findings, we conclude that a high index of suspicion for HEAML is clinically warranted for any hypervascular liver lesion, particularly in female patients without a background of chronic liver disease. Given the unreliability of imaging for definitive characterization, early consideration of percutaneous biopsy should be integrated into the diagnostic algorithm for such atypical cases. As complete surgical resection remains the standard of care due to the tumor’s malignant potential, this heightened clinical awareness is crucial for guiding appropriate management and avoiding potentially incorrect therapeutic pathways.

## Data Availability

The original contributions presented in the study are included in the article/supplementary material, further inquiries can be directed to the corresponding authors.
